# Recent Advances in Bioinspired Robot and Intelligent Systems

**DOI:** 10.3390/biomimetics11090678

**Published:** 2026-09-20

**Authors:** Guoniu Zhu, Yi Fang, Yang Yang

**Affiliations:** 1 College of Intelligent Robotics and Advanced Manufacturing, Fudan University, Shanghai 200433, China; 2 School of Automation and Intelligent Sensing, Shanghai Jiao Tong University, Shanghai 200240, China; apocalypse@sjtu.edu.cn; 3 Department of Electronic Engineering, The Chinese University of Hong Kong, Hong Kong SAR 999077, China; y.yang@cuhk.edu.hk

## 1. Introduction

Nature provides a rich source of principles for engineering systems capable of operating efficiently and robustly in complex environments [1]. Through biological evolution, organisms have developed highly effective solutions for locomotion, manipulation, sensing, adaptation, communication, and survival. Translating these principles into engineering has become an important pathway toward creating robotic systems with improved adaptability, efficiency, resilience, and autonomy [2,3,4].

The scope of bioinspired robotics has expanded substantially in recent years. Early efforts often emphasized the replication of biological morphology or motion, whereas contemporary research generally focuses on extracting the underlying functional principles of biological systems and transferring them to robotic structures, actuators, sensing strategies, control architectures, and learning systems [5,6,7]. Artificial intelligence further accelerates this transition by allowing robots to learn and adapt to environmental changes rather than relying exclusively on predefined models and controllers [8]. The field is therefore progressing from morphological imitation toward the integration of embodiment, perception, adaptation, and intelligence.

This Special Issue is composed of 12 papers that reflect this broadening research landscape. The contributions cover bioinspired structures and locomotion, humanoid manipulation and motion control, medical and human-interactive robotics, adaptive visual perception, swarm communication, and brain-inspired computation.

## 2. Contributions to the Special Issue

Several contributions demonstrate how biological structures and locomotion mechanisms can be translated into robotic embodiment. Fan et al. [9] developed PteroBot, a Pteromyini-inspired gliding robot designed for forest exploration. By employing deformable wing membranes, attitude modulation, and a bioinspired gliding strategy, PteroBot achieved a glide ratio of 2.02 while providing lower energy consumption and acoustic disturbance than conventional UAV-based approaches. The study illustrates how bioinspiration can simultaneously address mobility and ecological compatibility.

Wu et al. [10] investigated a bioinspired suction cup by translating the functional adhesion principles of the abalone muscular foot into an engineered suction structure. Instead of directly replicating biological morphology, the authors extracted two functional characteristics of the abalone foot, namely grooved surface structures and peripheral sealing, and translated them into an interconnected groove network and an annular sealing ring. Finite element analysis and experimental validation demonstrate improved interfacial pressure and frictional stress, together with reduced sliding, highlighting the value of functional abstraction in bioinspired adhesion design.

Zhu et al. [11] introduced a quadrupedal robotic platform featuring an actively articulated parallel torso with six degrees of freedom. Inspired by the flexible torsos of quadrupedal mammals, the active torso was coordinated with leg motion using a model predictive control framework accounting for changes in the center of mass and inertial properties. The results demonstrate the importance of torso–leg coordination for improving gait flexibility, stability, and locomotion efficiency.

Human musculoskeletal principles also motivate manipulation systems. Huang et al. [12] presented a fully decoupled tendon-driven humanoid arm based on antagonistic tendon actuation. A distinctive feature of this work is that joint-space decoupling is achieved primarily through mechanical architecture rather than control-based compensation. This study emphasizes the role of mechanical embodiment in simplifying control while retaining dynamic capability.

Cheng et al. [13] proposed a spatial parallel mechanism inspired by the human masticatory system. Their inverse dynamic model incorporates direct constraints and friction at higher kinematic pairs while using Euler parameters to represent end-effector rotation. The resulting formulation requires only approximately 23% of the computational time of the corresponding Euler angle-based model, providing a basis for efficient real-time control of biologically congruent mechanisms.

Bioinspired and human-centered design is also represented in medical robotics. Silva-Garcés et al. [14] developed a neurosurgery-assisting device that employs a 3-RPS parallel architecture. With its design based on the geometry and workspace of the human head, the system integrates linear actuation with current, acceleration, and force sensing for cannula positioning and insertion. Prototype experiments demonstrate the feasibility of the proposed architecture, illustrating how anatomical constraints can directly inform robotic mechanism design.

Human biological motion can further serve as an intuitive control interface. Drăgoi et al. [15] suggested a vision-based teleoperation framework that enables users to control an articulated robotic arm through natural hand movements and gestures captured by a conventional camera. The work demonstrates the potential of accessible vision-based interfaces for human–robot interaction without specialized wearable sensing equipment.

Beyond morphology and mechanisms, several contributions focus on learning and perception. Jiang et al. [16] explored the learning of sitting and standing-up behaviors in humanoid robots through a progressive reinforcement learning strategy. The first stage encourages task exploration, whereas the second stage refines the resulting trajectories through adaptive tracking and additional motion constraints. The approach achieved simulation success rates of 99.8% for sitting and 99.5% for standing while producing smoother and safer motions, demonstrating the potential of progressive learning strategies for complex, non-periodic humanoid behaviors.

Lee and Kang [17] addressed robust ego-motion estimation in dynamic scenes by combining Extended Kalman Filter-based state estimation with an improved 1-point RANSAC strategy. Inspired by adaptive biological visual perception, the approach treats dynamic visual information as potentially useful rather than simply rejecting all moving landmarks as outliers. By combining static and dynamic landmarks with dynamic-object estimation, the framework provides a biologically motivated approach to robust ego-motion estimation in complex environments.

Bioinspiration also extends to collective intelligence. Starbuck et al. [18] investigated visual communication in UAV swarms by drawing inspiration from biological communication strategies such as bee waggle dancing and animal visual displays. A generative AI-based translation layer produced motion- and LED-based visual signals for communication in radio frequency-denied environments. Motion-based communication achieved particularly high levels of clarity and expressiveness, whereas LED-based and multimodal signaling revealed a trade-off between signal richness and interpretability. This work broadens bioinspiration from physical robot design to decentralized communication and multi-agent behavior.

At the level of cognitive modeling, Vallverdú and Rius [19] presented a quantum-inspired paradigm for brain emulation. The framework derives a Schrödinger-like representation using Nelson’s stochastic mechanics and explores its implementation through quantum simulation methods. Rather than claiming that the brain itself performs quantum computation, their study uses quantum-inspired mathematical tools to model stochastic and nonlinear neural dynamics, extending the conceptual scope of biologically inspired intelligent systems.

Finally, Jung et al. [20] proposed a systematic overview of how artificial intelligence is reshaping control and adaptation in biomimetic robotic systems. By examining locomotion, sensory perception, cognitive learning, and related biomimetic functions, their review illustrates how machine learning, deep learning, reinforcement learning, and evolutionary methods are integrating with bioinspired robotic platforms. It therefore provides a useful overarching framework for many of the research directions represented throughout this Special Issue.

## 3. Conclusions

The 12 collected contributions demonstrate the breadth of contemporary bioinspired robotics and intelligent systems. They span biological inspiration from the mechanical to the cognitive and collective levels, including adhesion, musculoskeletal structures, locomotion, manipulation, human–robot interaction, perception, learning, communication, and neural computation.

A common theme across these studies is that contemporary bioinspiration emphasizes the reproduction of functional principles, rather than the mere imitation of biological appearance. Biological structures, for example, can endow robotic bodies with advantageous mechanical properties, while biological learning and perception can inspire adaptive control strategies. Likewise, biological communication and neural processes can provide new paradigms for collective and artificial intelligence. The convergence of these directions is driving bioinspired robotics toward more integrated embodied systems in which morphology, sensing, control, and intelligence are closely coupled.

We sincerely thank all of the authors for their valuable contributions to this Special Issue and the reviewers for their time, expertise, and constructive comments. We also thank the editorial staff of *Biomimetics* for their support throughout the editorial and publication process. We hope that this collection will stimulate further interdisciplinary research at the intersection of biology, robotics, control, artificial intelligence, and intelligent systems.
